# Image-guided intramyocardial cell injection: putting a puzzle piece in the right place

**DOI:** 10.1007/s12471-014-0608-y

**Published:** 2014-10-21

**Authors:** A. A. Ramkisoensing, D. E. Atsma

**Affiliations:** Department of Cardiology, LUMC, 2300 RC Leiden, the Netherlands

Despite significant advances in the management of acute myocardial infarction, it remains a major cause of morbidity and mortality. After myocardial infarction, viable myocardium is lost and is replaced by fibrotic tissue. This increases the tensile strength of the infarcted myocardium preventing cardiac rupture. However, ultimately it impairs the contractile capacity of the heart leading to heart failure.

For several years, cell transplantation has been employed in the damaged heart of patients with end-stage heart failure. The aim of cell transplantation is to improve cardiac function. Indeed, to this point clinical trials show a modest therapeutic effect of cardiac cell therapy on cardiac function. The positive effects of cell therapy are believed to be mainly mediated by paracrine factors excreted by the transplanted cells. The role of direct cardiomyogenic or angiogenic differentiation of the transplanted cells that have been used in the clinical trials is thought to be small. Furthermore, pre-clinical studies have shown that only low percentages of injected cells survive and engraft in the infarcted myocardium. This finding could explain the modest effects that have been observed in both pre-clinical and clinical studies. Several studies are therefore aiming to improve stem cell survival and engraftment (e.g. by using biomaterials). Besides improving cell survival and engraftment, a better selection of the location of cell injection may also influence the therapeutic effect of such therapy. In the study by Van Slochteren et al. a 3D Cardbox image registration toolbox is used which makes it possible to standardise the transplantation location.[1] In this study, the researchers aimed to transplant stem cells in the 50 % infarct transmurality border zone. It is expected that in this area the stem cells are able to exert their therapeutic effects, while not being deprived of oxygen and nutrients. Indeed, it is known that the physiological niche of bone marrow-derived mesenchymal stem cells, frequently studied for cardiac cell therapy, has a low oxygen tension. Furthermore, several studies have shown that hypoxia enhanced the tissue regenerative potential of bone marrow-derived mesenchymal stem cells.[2, 3] Injection of stem cells in the correct location is therefore of importance to obtain the maximum therapeutic effect, which makes the results of this study highly relevant.

The positive effect of cell therapy is also thought to be dependent on cell-to-cell contact. In a recent study it was shown that gap junctional coupling, which is essential in electrochemical communication between cardiomyocytes, is also a key factor in inducing cardiomyogenic differentiation of mesenchymal stem cells. After downregulation of connexin43, the major gap junction protein in the myocardium, cardiomyogenic differentiation of the stem cells was prevented. By overexpressing connexin45 and thereby restoring the ability to respond to cardiomyogenic stimuli provided by neighbouring cardiomyocytes, the cardiomyogenic differentiation potential of the mesenchymal stem cells was restored.[4] The injection at location defined by the study by Van Slochteren et al.[1] makes interaction with cardiomyocytes possible and may therefore increase the therapeutic potential of cardiac cell therapy. To our knowledge, cardiomyogenic differentiation of mesenchymal stem cells that were only adjacent to cardiac fibroblasts was never observed in pre-clinical studies. Whether the excretion of paracrine factors that are involved in regeneration of the infarcted myocardium also depends on the interaction of cardiomyocytes with stem cells, has not yet been investigated [5].

By employing techniques that allow more precise and standardised selection of the target location for cell transplantation, the retention of stem cells in the targeted area may also improve by favouring survival and engraftment. An increase in the retention of stem cells may lead to an increase in the occurrence of arrhythmias. The occurrence of arrhythmias was not studied in the study by Van Slochteren et al.[1] While they show an elegant way to improve injection at the correct location, the engraftment pattern of the stem cells is not controlled by using the 3D Cardbox. In a recent study by Askar et al., the effects of these abovementioned factors, engraftment number and pattern, were shown to be related to the occurrence of arrhythmias.[6] Further investigation is therefore needed to fully understand the role of the 3D Cardbox in optimising cardiac regeneration by stem cell transplantation.
